# Perceived Needs Among Asylum Seekers in Sweden: A Mixed Methods Study

**DOI:** 10.3390/ijerph17144983

**Published:** 2020-07-10

**Authors:** Karin Hugelius, Maya Semrau, Marie Holmefur

**Affiliations:** 1 School of Health Sciences, Orebro University, 70182 Orebro, Sweden; marie.holmefur@oru.se; 2 Centre for Global Health Research, Brighton and Sussex Medical School, Brighton BN1 9PX, UK; m.semrau@bsms.ac.uk

**Keywords:** asylum seekers, needs assessment, social support, mental health, mixed method

## Abstract

The health and well-being of asylum seekers in high-income countries is a concern from both individual and community perspectives. This study aims to describe the perceived needs of adult asylum seekers in Sweden. A mixed methods study was conducted that combined a non-randomized descriptive cross-sectional assessment of perceived serious needs using the Humanitarian Emergency Settings Perceived Needs Scale (HESPER) Web with 85 adult asylum seekers and focus group discussions with 14 adult asylum seekers in Sweden. Descriptive and comparative statistics were used for the quantitative part, and thematic analysis for the qualitative part. The total number of perceived serious needs reported by respondents ranged from zero to 13 needs per person with a mean of four needs (SD 2.71). The most commonly perceived serious needs were related to income or livelihood, separation from loved ones, being displaced from home, distress, and concerns about accessing adequate health care services. Many of the perceived needs appeared to be related to experiences of being dependent, in limbo, and vulnerable. Addressing people’s current perceived needs can contribute to resilience and well-being and therefore should be considered in health care systems that cater to immigrants.

## 1. Introduction

The total number of people living in a country other than their country of birth is increasing due to conflicts and humanitarian emergencies. In total, 68.5 million people were forcibly displaced by persecution, conflicts, violence, or human rights violence, in 2017 [1]. From a global perspective, most of the refugees are internal, displaced within their own country, but some also immigrate to other countries temporarily or permanently. Asylum seekers, individuals seeking international protection but whose claim for refugee status has not yet been determined, can be considered to be a vulnerable population given factors such as immigration status, the upheaval of moving from one’s homeland to a very different society, socioeconomic factors, use and access to health care services, and exposure to trauma [2]. The health and well-being of asylum seekers is a concern both from an individual perspective and a national health view. Limited access to health care services, an influx of neglected disease, and a restructuring of the health care services to assist immigrants, refugees, and asylum seekers can impact the overall health of a nation [2,3]. In recent years, the Swedish immigrant population has increased rapidly. In 2017, about 25,500 people sought asylum in Sweden, most of them from Syria, Iraq, and Iran [4]. Previous studies have described several health problems among asylum seekers in high-income countries, including both physical and mental health problems such as post-traumatic stress and depression [5,6]. Many asylum seekers and refugees in Sweden have also experienced a poor quality of life [7,8].

The need to understand health and well-being among asylum seekers and other immigrants in high-income countries is, therefore, of great interest, but there are limited studies reporting on their needs [3]. In needs assessments, the involvement of the affected people is crucial to ensure a proper response based on their actual perceived needs and not estimated needs [9]. In this study, perceived needs are defined as needs expressed by members of the affected population themselves [10]. Therefore, they are the needs and perceived problems for which people would likely want help and a good indicator when assessing health and well-being [10]. In addition, it has been suggested that to promote mental health and increased life quality, it is essential to evaluate perceived current problems instead of just focusing on past traumatic events [3,6,11]

However, the authors have found no study of perceived needs among asylum seekers in Sweden or other similar settings or countries. Therefore, this paper aims to describe the perceived needs of adult asylum seekers in Sweden.

## 2. Methods

An explanatory sequential mixed methods study design [12] was used. The quantitative part consisted of a descriptive, cross-sectional, web-based survey, using data collected for a psychometric evaluation of the Humanitarian Emergency Settings Perceived Needs Scale (HESPER) Web, which has been presented elsewhere [13]. The qualitative part consisted of focus group interviews analysed using a theoretical thematic analysis [14]. A qualitative data collection was conducted for this study, and therefore is considered to be primary data. The two data collections were made separately and with different study samples.

### 2.1. Quantitative Part

#### 2.1.1. The HESPER Web Survey

The HESPER scale [15] is an interview-based instrument developed to assess the perceived needs of people affected by emergencies, disasters, or other humanitarian situations. It has been used for needs assessments in both humanitarian response and research studies and found to be a valid and scientifically robust instrument to assess perceived needs [15,16]. The HESPER Web used in this study is a self-administrated, web-based survey version of the original HESPER scale. The HESPER Web consists of 26 items on perceived needs, ranging from access to drinkable water to education for children, physical or mental health problems, and perceived sense of security. Ratings are made by categorising the need as “serious need” or “no serious need”. The survey also asks the respondent to prioritise their three most important serious needs. The survey further requests the respondent’s gender, age, current location, and country of origin. The development and psychometric evaluation of the HESPER Web has been reported elsewhere [13].

#### 2.1.2. Study Sample and Data-Collecting Procedures

A non-randomised, voluntary study sample of 85 adult asylum seekers in one county in Sweden participated in this study. The county is comprised of approximately 300,000 inhabitants in both urban and rural areas. Data were collected between April and June 2018. During that period, the region had 1877 registered adult asylum seekers [5].

To take part in the study, participants had to be at least 18 years old, be asylum seekers, or have received a temporary asylum decision in Sweden and have access to a smartphone, tablet, or computer with Internet access. Sufficient English skills were also required to complete the survey. Study participants were recruited through information meetings at social activities arranged by the local Red Cross or the local church and by official postings in asylum seekers’ designated living areas. All data collection was carried out in English. Data were collected as part of a study that aimed to evaluate the psychometric characteristics of the HESPER Web [13]. The data used in this study consisted of the individual answers given when the study participants answered the HESPER Web survey for the first time, either as part of an alternative forms evaluation or as the first data collection in a test-retest evaluation [13]. The study participants completed the survey anonymously by mobile phone, tablet, or computer.

#### 2.1.3. Data Analysis

To analyse the data, descriptive statistics with the mean, standard deviation (SD), and range (for age) were used to describe the study sample characteristics and the number of perceived needs. Differences between gender, age, or country of origin were compared with Chi2 or Student’s t-test. Data analysis was conducted in SPSS (IBM Corp. Released 2016. IBM SPSS Statistics for Windows, Version 24.0. Armonk, NY: IBM Corp) by two of the researchers (K.H. and M.H.) and verified by an independent health statistician. A *p*-value of 0.05 was considered statistically significant in all analyses.

### 2.2. Qualitative Part

#### 2.2.1. Study Sample and Data-Collecting Procedures

For the qualitative study, focus group discussions with 14 adult asylum seekers or persons with a newly issued temporary permit to stay in Sweden participated. Data collection was carried out in March 2019 in the same region as for the quantitative data collection. Participants were recruited by oral invitations at a local language-training café. Three focus group discussions were conducted, with four participants in each of the first and second focus groups and six in the third focus group. The focus groups were conducted on different days of the week, and the participants could choose freely when to participate. Two of the focus groups (one with four and one with six participants) consisted of both male and female participants, and the third group with four people comprised only female participants. No selection was made to form the groups beforehand. No study participant taking part in the focus group discussions had participated in either the HESPER survey. The focus group discussions were led by one of the researchers (K.H.), assisted by a male volunteer from the local Red Cross branch, who was himself a former asylum seeker and familiar with the study participants from voluntary services at the language-training café. The researcher had a professional background as a nurse with experience conducting qualitative research in humanitarian and emergency contexts. The researcher had no previous knowledge of or personal relation with the study participants. The focus groups lasted between 15 and 55 min. They began with a short presentation of the quantitative results, showing a diagram of the most reported perceived needs, based on the HESPER survey. This was followed by a discussion of perceived needs in general and in relation to the participants’ current situations. The same structure was used for all interviews, but no specific interview guide was used. The focus groups were conducted in Swedish and audio recorded, with the researcher taking notes during and after each discussion.

#### 2.2.2. Analysis

The focus group discussions were transcribed verbatim and analysed using a theoretical, semantic thematic analysis [14] approach. After reading through the texts and making initial notes on immediate impressions from the data, the researcher who conducted the interviews extracted and coded meaning units that corresponded with the study’s aims, using the HESPER items as a theoretical guidance. Thereafter, the codes were grouped into potential themes. During this process, thematic maps were also used to inform the analysis process, in accordance with the method used [14]. The themes were reflected on in relation to the coded extracts, the entire texts, and the notes from the interviews and of immediate impressions. Finally, all the themes identified were named. The analysis process was performed by the researcher who had conducted the interviews and was verified by another author (M.H.), who also read all the interviews in their entirety.

#### 2.2.3. Interpreting and Merging the Quantitative and Qualitative Data

The quantitative and qualitative datasets were collected and analysed separately; and in accordance with the explanatory sequential mixed methods study design [12], the analysis of the qualitative data was influenced by the quantitative instrument used. As a final step, the results of both data were merged and interpreted as a comprehensive summary of the total findings [12].

#### 2.2.4. Ethical Considerations

Ethical approval from the Swedish Ethical Review Authority (2017/481) was obtained before data collection started. In the study information, it was clearly stated where participants should turn to if they experienced any acute needs. All participants were given full information about the study in writing (Swedish and English) and orally (Swedish, English or Arabic) before signing informed consent forms (digitally in the web survey or on paper for the focus groups interviews).

## 3. Results

In total, 99 adult asylum seekers participated in the study. Of these, 85 participated in the web-based survey and 14 in the qualitative focus group discussions. The demographic characteristics for the study participants are listed in Table 1.

### 3.1. Quantitative Results

#### 3.1.1. Total Number of Serious Needs

The total number of serious needs reported in the HESPER Web varied from zero (5% of study participants) to 13 (2%) needs per person, with a mean of four needs (SD 2.71). There was no significant difference between men and women in terms of the total number of needs (χ^2^ 11.039, *p* = 0.36) and there was no association between age and the total number of needs (Pearson correlation 0.009, *p* = 0.957) or country of origin and the total number of needs (Pearson correlation 0.007, *p* = 0.858).

#### 3.1.2. Item-Level Perceived Serious Needs

The most frequently perceived serious need was income or livelihood. Thereafter, separation from family members, being displaced from home, and distress were reported by several study participants (see Table 2). For some of the items, there were differences between genders. More female study participants reported a serious need for health care services than male participants. Distress, support from others and needs due to being displaced from home were also reported more often by female participants. A lack of respect was reported more often by male participants (see Table 2). Income or livelihood was most frequently prioritised as the “most serious” need by study participants (reported as the priority for 21% of all study participants), followed by separation (12%) and physical health (9%).

#### 3.1.3. Dropouts from the HESPER Web Survey

The internal dropout on an item level varied between zero and 16 persons. The questions with the most missing data concerned current location (16 missing answers, 18%) and if technical problems had occurred (12 answers missing, 14%). Among the perceived problem items, the dropouts varied from zero to four missing answers per item. Since the dropout level was considered to be low, no further dropout analysis or measures were undertaken.

### 3.2. Qualitative Results

The qualitative analysis resulted in the following three themes: being dependent, being in a limbo state, and being vulnerable. All the codes found in the qualitative data could be associated with an original HESPER item. The theme “being dependent” described experiences of being in a physical location and context that were not self-chosen. The theme included the codes “how aid is provided”, “place to live in”, “income or livelihood”, and “moving between places”. On the one hand, study participants expressed feelings of gratitude for all the services given, including being accepted into the country and provided with a place to stay, daily stipends, and basic services. As one female participant said, “If I had the choice to choose entirely by myself, I would have chosen to live somewhere else. But this is better than the alternative, so I accept it” (Focus group 1).

On the other hand, being dependent on the system and others’ decisions and not being satisfied with one’s situation or others’ decisions created feelings of being trapped and frustrated. One male participant observed, “I think that most adults would like to be able to earn one’s living by themselves. I think it is a general need among most of us. I don´t feel very proud of being dependent on others” (Focus group 1).

The theme “being in a limbo state” was characterized by uncertainty related to both legal statuses, including waiting for a decision on the permit to stay, and uncertainty regarding the future in general. “Being in a limbo state” included the codes “respect”, “too much free time”, and “lack of information”. Not being fully included in society, not having a job, or being allowed to work or study created problems related to too much free time, which also affected the participants’ well-being. One female participant noted, “Because I have nothing to do, I think my mind reminds me a lot of things that are not good for me” (Focus group 2).

Feelings of losing one’s integrity and not being treated as a human were also expressed, “No one actually cares about who you are” (Focus group 3, female participant). Furthermore, the participants experienced a lack of information, which was mainly related to the need to understand the Swedish society, such as where to turn for specific services or information about the asylum process.

The theme “being vulnerable” was related to both physical and mental aspects of well-being. It included the codes distress, physical health, separation from family, being displaced from home, safety, access to health care. and support from others. Participants expressed how they mourned their old life, regarding both practical circumstances and their role in society. One female participant said, “When you are here, you are safe. I like it here. But in the same time, I miss my country, I miss my old house, even if I know it is not there anymore. My old life does not exist, but I still miss it” (Focus group 2).

Missing loved ones who were deceased, left behind, or asylum seekers in another country or whose status was unknown created longing, sadness, depression, and loneliness. General worries about the future made the participants feel weak and vulnerable. One participant said, “I think that I´m a weak person right now. I often feel sad” (Focus group 1, male participant).

The participants expressed how mental pressure or traumatic experiences caused both mental health problems, such as anxiety and nightmares, and physical health problems, such as bodily pain or dizziness. One female participant observed, “Having nothing to do, that is tough for my soul. And when my soul is suffering, my body is suffering” (Focus group 2).

Some participants also mentioned specific health problems, such as high blood pressure and tuberculosis. Experiences of not trusting the health care services to provide necessary treatment or of health professionals neglecting to report health problems were expressed and worsened the feelings of being vulnerable.

#### Summarized Understanding of the Qualitative and Quantitative Data

All codes identified in the qualitative analysis could also be found among the ten most frequently reported needs in the HESPER survey. The most reported need, a lack of income or livelihood, in combination with being dissatisfied with how aid was provided, being in a location that was not self-chosen and moving between places was related to an experience of being dependent on a foreign system and others’ decisions. This caused frustrations and negative feelings of being trapped. At the same time, study participants expressed thankfulness to their new country.

The experience of a lack of respect, which was more frequently expressed by male, rather than female, participants, was related to the experience of being in limbo, having too much free time, and lacking information. This caused feelings of uncertainty and being excluded from the society. Having too much free time and a lack of information also caused feelings of uncertainty and being excluded from society. Both distress and physical health symptoms, as well as a lack of access to or mistrust of available health care services, were reported, especially among female participants. In the interviews, separation from loved ones, being displaced from home, lack of social support, and safety issues were experienced as having a negative impact on health and well-being. These reported needs made the participants feel vulnerable.

## 4. Discussion

This study has shown that a non-randomized sample of asylum seekers in Sweden experienced a variety of needs in their current situation. The needs reported most frequently were related to income or livelihood, separation from loved ones, being displaced from home, distress, and concerns about accessing adequate health care services. The perceived needs can be related to the experiences of being dependent, being in limbo, and being vulnerable.

The most prominent need in this study was lack of income or livelihood, which was closely linked to the experiences of dependency and being in limbo, expressed as, for example, having too much free time and not contributing to society. Lack of employment or a meaningful occupation was similarly found to be a major problem among asylum seekers or forced migrants in other studies [16,17,18]. This shows an acute need for interventions targeting the occupational needs of this population, whether with paid employment, volunteer work, or other means to satisfy individual asylum seekers’ need for meaningful occupation.

Asylum seekers, in general, have complex health profiles, including physical and mental health aspects. Accordingly, access to health care services was stressed as an important need among the study participants, particularly female participants. Previous studies have highlighted several barriers to asylum seekers accessing health care services, including legal aspects, mistrust, language and cultural factors, lack of awareness, stigma, and negative attitudes towards and by providers [19,20]. Elements of mistrust and feelings of being neglected were also expressed by participants in the focus group discussions in this study. More female than male participants perceived that there was a serious need for health care services, although the exact reason for this result is not known. There is a lack of evidence regarding how best to identify and deter the negative reproductive health outcomes of resettling refugee women, which are associated with their migration experience as compared with their non-refugee counterparts [21]. However, medical services for emergencies, including maternal health care and other reproduction care, are freely available to asylum seekers in Sweden. Therefore, female participants’ assertion of problems related to access to medical care deserves further exploration.

Asylum seekers in high-income countries are at increased risk for mental health problems as compared with non-asylum seekers [7,22]. Negative experiences such as moving to a very different society, economic difficulties, perceived discrimination, and feelings of poor control over one’s life can be associated with a higher prevalence of distress [23,24]. The wait for an asylum decision entails continued uncertainty, and a clear majority of asylum seekers live in a high-pressure situation, socially and economically [25]. Needs secondary to not having a job were emphasized in both the survey and the focus groups. Standing outside the labor market means that your possibilities for earning your living, contributing to society, and being active during the day are limited. Furthermore, in this study, distress was reported by 34% of the participants. This is in line with previous studies of the prevalence of mental health issues among asylum seekers and other immigrants in Sweden [7,26]. The qualitative discussions added perspectives on the content of the distress, including mourning of lost life contexts, loss of loved ones, and feelings of loneliness, sadness, weakness, and depression. Another possible component of the prevalence of distress could be the lack of access to mental health services. Historically, mental illness and distress among humanitarian populations have been blamed mainly on experiences of potentially traumatic events in the country of origin or during the emergency [9]. However, several studies have indicated that daily stressors partially mediated the relationship between war exposure and mental health problems in immigrant populations [26]. One study called for increasing the focus on ongoing stressors arising from current perceived problems instead of past traumatic events in humanitarian populations and suggested that a positive post-emergency environment could mediate potentially traumatic and negative experiences [27,28].

Therefore, a needs assessment focusing on the perceived current situation should be of interest to every asylum-seeker host organization and health care system providing care for immigrants, including those in high-income countries. The HESPER Web can be a useful tool for such assessments and contributes valuable information about the health and well-being of a vulnerable population. Supportive activities, such as mobilizing social support, community mental health interventions, and occupational interventions, can be initiated if a perceived need is indicated. Further research on the effects of interventions, such as community-based mental health interventions, for asylum seekers and other refugees in high-income countries, however, is needed. The HEPSER Web could be used to evaluate such interventions in longitudinal studies.

## 5. Limitations

This study had several limitations. The quantitative data relied on a non-randomized sample of asylum seekers in a specific county in Sweden, and therefore the generalizability of the result could be limited. However, knowledge of the perceived needs and health among asylum seekers in high-income countries is strongly needed [5], and this study still contributes to the development of such knowledge. Because the system and services for asylum seekers in Sweden are national, there is no reason to believe that the results could not be generalized to other parts of the country. Furthermore, some participants in the quantitative part of the study were fluent English speakers, and some were not. As the HESPER Web survey was in English, language ability could have influenced their answers to the questions. Using a web-based method for data collection limits the ability to explain and clarify questions, which can cause a risk of dropout. This study was based on secondary data from a psychometric evaluation of the HESPER Web. Using web-based surveys has several advantages, such as a reduced number of internal dropouts and processing errors, as well as quicker data collection and analysis; they are also often a more economical alternative to other types of surveys [28]. A comparison of the means of reported needs among those study participants who participated in all parts of the psychometric evaluation and those who did not participate in the second step of the alternate or test-retest evaluation showed no significant difference in the mean number of reported needs. The HESPER Web is designed for descriptive analysis, and therefore the possibilities for conducting regression models are limited due to mathematical concerns.

The focus group participants were recruited from a language café in a specific town, which must be taken into consideration when interpreting the results. However, the focus groups included both male and female participants of different ages, which enriched the data. The focus group discussions were conducted in Swedish, precluding both the study participants and the non-Swedish-speaking researcher from participating fully in the analysis process. Language ability could also have influenced the short duration of one of the focus group discussions. Using focus groups instead of individual interviews can increase the participants’ sense of security and comfort and is recommended for studies that stress strong emotions, as the group can be perceived as supportive [29]. However, participants in focus groups cannot be assured complete confidentiality the same way as in individual interviews, but in this study, all participants were requested to handle the information they received during the focus group with respect. Finally, the items in the HESPER Web were used to influence the analysis of the qualitative data, which is part of the chosen explanatory sequential mixed methods study design; however, this could have influenced the interpretation of the qualitative data.

## 6. Conclusions

Perceived needs among the adult asylum seekers in this study were related mainly to income or livelihood, separation from loved ones, being displaced from home, distress, and concerns about accessing adequate health care services. These needs lead to an experience of being dependent, being in a limbo state, and being vulnerable. Having a daily occupation seems to be an important step for the well-being of this population, and therefore should be considered as health promoting intervention.

Focusing on the assessment of and addressing the current perceived needs of asylum seekers can contribute to their health and well-being by drawing attention to the perceived needs instead of previous experiences. Therefore, the perceived needs should be considered by professionals responsible for the health care and social integration of asylum seekers. The HESPER Web can be recommended for such assessments.

## Figures and Tables

**Table 1:** Demographic characteristics of study participants.

	Quantitative Part	Qualitative Part
Study participants *n* = 99	*n* = 85	*n* = 14
Gender, *n* (%) Male	45 (53%)	4 (29%)
Female	40 (47%)	10 (71%)
Age, mean (SD) range	32 (8.7) 19–71	30 (6.5) 22–41
Country of origin, n (%)		
Eritrea	24 (29%)	11 (78%)
Afghanistan	16 (19%)	0 (0%)
Syria	13 (16%)	3 (21%)
Somalia	12 (14%)	0 (0%)
Other/stateless/	20 (22%)	0 (0%)
Do not want to say		

*n*, number of persons; SD, standard deviation.

**Table 2:** Perceived serious needs, item by item.

Item	Total Persons Reporting a Need *n* (%)	Men Reporting a Need *n* (%)	Women Reporting a Need *n* (%)	Differences Between Gender *p*-Value ^a^
N	85	45	40	
Income or livelihood	50 (58.8)	29 (64.4)	21 (52.5)	0.280
Separation from family members	41 (48.8)	20 (44.4)	21 (53.8)	0.512
Being displaced from home	39 (45.9)	15 (33.3)	24 (60.0)	0.017 *
Distress	29 (34.1)	9 (20.0)	20 (50.0)	0.006 *
Too much free time	25 (30.1)	16 (37.2)	9 (22.5)	0.160
Health care	24 (28.2)	7 (15.6)	17 (42.5)	0.008 *
Support from others	19 (22.4)	4 (8.9)	15 (37.5)	0.002 *
Moving between places	22 (26.5)	17 (37.8)	5 (13.2)	0.013 *
Place to live in	15 (17.6)	8 (17.8)	7 (17.5)	1.000
Physical health	15 (17.6)	9 (20.0)	6 (15.0)	0.582
The way aid is provided	13 (16.0)	10 (23.3)	3 (7.9)	0.074
Respect	13 (15.3)	11 (24.4)	2 (5.0)	0.016 *
Information	10 (12.0)	6 (14.0)	4 (10.0)	0.740
Other	8 (11.4)	0 (0.0)	8 (23.5)	0.002 *
Law and justice in your community	4 (5.1)	4 (9.5)	0 (0.0)	0.120
Waiting for asylum decision	5 (5.9)	0 (0.0)	5 (12.5)	0.020 *
Care for people in your community who are on their own	3 (4.2)	1 (2.6)	2 (6.1)	0.594
Mental illness in your community	2 (2.9)	2 (5.4)	0 (0.0)	0.496
Care for family members	2 (2.6)	0 (0.0)	2 (5.3)	0.493
Safety	2 (2.4)	0 (0.0)	2 (5.0)	0.218
No job	2 (2.4)	0 (0.0)	2 (5.0)	0.218
Alcohol or drug use in your community	1 (1.3)	1 (2.5)	0 (0.0)	1.000
Clothes, shoes, bedding, or blankets	1 (1.2)	0 (0.0)	1 (2.5)	0.471
Toilets	0 (0.0)	0 (0.0)	0 (0.0)	-
Keeping clean	0 (0.0)	0 (0.0)	0 (0.0)	-
Education for your children	0 (0.0)	0 (0.0)	0 (0.0)	-
Safety or protection from violence for women in your community	0 (0.0)	0 (0.0)	0 (0.0)	-
Total number of needs, mean (SD) (range)	3.98 (2.71) (0–13)	3.76 (2.59) (0–10)	4.23 (2.86) (0–13)	0.429 *^b^*

*a, p* calculated with Chi-2 test; *b, p* calculated with Student’s t-test; * indicates a significant result.
